# Thioredoxin protects mitochondrial structure, function and biogenesis in myocardial ischemia-reperfusion via redox-dependent activation of AKT-CREB- PGC1α pathway in aged mice

**DOI:** 10.18632/aging.104071

**Published:** 2020-10-13

**Authors:** Jaganathan Subramani, Venkatesh Kundumani-Sridharan, Kumuda C. Das

**Affiliations:** 1 Department of Internal Medicine, Texas Tech University Health Sciences Center, Lubbock, TX 79430, USA

**Keywords:** aging, heart, ischemia-reperfusion, thioredoxin, mitochondria

## Abstract

Aging is an independent risk factor for cardiovascular diseases, such as myocardial infarction due to ischemia-reperfusion injury (I/R) of the heart. Cytosolic thioredoxin (Trx) is a multifunctional redox protein which has antioxidant and protein disulfide reducing properties. We hypothesized that high levels of Trx will protect against multifactorial disease such as myocardial infarction due to I/R injury in aged mice. Aged mice overexpressing human Trx (*Trx-Tg*), mice expressing redox-inactive mutant of human Trx (*dnTrx-Tg*) and non-transgenic litter-mates (*NT)* were subjected to I/R (60/30 min), and cardiac function, mitochondrial structure and function, and biogenesis involving PGC1α pathway were evaluated in these mice. While aged *Trx-Tg* mice were protected from I/R-induced reduction in ejection fraction (EF) and fractional shortening (FS), had smaller infarct with decreased apoptosis and preserved mitochondrial function, aged *dnTrx-Tg* mice showed enhanced myocardial injury and mitochondrial dysfunction. Further, *Trx-Tg* mice were protected from I/R induced loss of PGC1α, ACO2, MFN1 and MFN2 in the myocardium. The *dnTrx-Tg* mice were highly sensitive to I/R induced apoptosis. Overall, our study demonstrated that the loss of Trx redox balance in I/R in aged *NT* or *dnTrx-Tg* mice resulted in decreased PGC1α expression that decreased mitochondrial gene expression with increased myocardial apoptosis. High levels of Trx, but not mitochondrial thioredoxin (Trx-2) maintained Trx redox balance in I/R resulting in increased PGC1α expression via AKT/CREB activation upregulating mitochondrial gene expression and protection against I/R injury.

## INTRODUCTION

Aging is an independent risk factor for cardiovascular disorders including ischemic heart diseases [1]. Aged hearts are more likely to fail in ischemia-reperfusion injury (I/R) compared to younger hearts, as endogenous antioxidative capacity declines with aging. Aging is also associated with oxidative protein modifications and loss of function [2]. However, interventions with antioxidants have not been very effective against I/R injury suggesting that additional mechanisms are involved in high incidence of age-dependent cardiovascular diseases. In this regard it is established that reactive oxygen species (ROS), which are produced during the reperfusion of the myocardium cause extensive damage to the affected heart tissue [3]. Although uncoupled endothelial nitric oxide synthase (eNOS), xanthine oxidase (XO), NADPH oxidases (Nox) and mitochondria are implicated as sources of ROS, mitochondria-mediated ROS release is of critical importance in cardiomyocyte death via apoptosis or necrosis [4–6].

We have previously demonstrated that aged heart mitochondria respire at a lower rate compared to young mice, and metabolic demand on oxidation of mitochondrial fuels are decreased with age [7]. The energy mitochondria generate by oxidative phosphorylation is required for normal heart function. Paradoxically, mitochondria are also significant source of the ROS generated during normal respiration [8]. Altered mitochondrial structure and function in the aging process have been shown to aggravate I/R-mediated mitochondrial dysfunction [9]. Age-related defects in various mitochondrial ETC complexes combined with increased mitochondrial fission and decreased fusion process impairs overall mitochondrial capacity in the aging, which is further deteriorated during I/R [10, 11]. Mitochondrial biogenesis led by peroxisome proliferator-activated receptor γ (PPARγ) coactivator-1α (PGC1α), is an important member of transcriptional coactivators family [12], regulates mitochondrial energy metabolism and cardiac function [13]. PGC1α is regulated by transcription and posttranslational modifications, such as phosphorylation, acetylation and methylation. In addition to promoting mitochondrial biogenesis, PGC1α has also been shown to be involved in the induction of several ROS detoxifying enzymes [14]. Ectopic expression of PGC1α in muscle cells induces superoxide dismutase-2 and glutathione peroxidase I, both of which are involved in removal of ROS.

Trx is a small (12 kDa) redox protein that is an electron donor for ribonucleotide reductase for the synthesis of deoxyribonucleotides, a rate-limiting step in DNA replication [15]. Trx is also an electron donor for peroxiredoxins, which detoxify peroxides [16]. Thioredoxin reductase-1 (TrxR1) uses reducing equivalents form NADPH and transfers electrons to recycle oxidized Trx produced in redox reactions to reduce Trx [17]. Trx scavenges hydroxyl radicals, quenches singlet oxygen, and induces mitochondrial SOD2 [18, 19]. A mitochondrial thioredoxin-2 (Trx-2) is present in the mitochondria of cells. Although active center cysteines of Trx is preserved in Trx-2, it lacks the additional structural cysteines that are present in Trx. Trx-2 has been shown to diminish mitochondrial ROS [20], and decreases myocardial apoptosis by reducing mitochondrial ROS [21]. We have previously shown that Trx regulates MAP Kinase Kinase-4 (MKK4) activation via redox regulation resulting in sequential activation of NFκB and AP-1, which regulates SOD2 expression in endothelial cells [19]. In addition, a major function of Trx includes regeneration of –SH group enzymes and proteins, which are inactivated by oxidation [15]. Thus, Trx not only is a radical scavenger or inducer of SOD2, but also converts oxidized proteins to their native state through its disulfide reductase properties. We have earlier demonstrated that high levels of Trx prevent I/R injury in adult mouse heart by eNOS deglutathionylation [22] and prevent age –related hypertension involving vascular mechanisms in mice [23]. Additionally, a previous study has demonstrated decreased post-myocardial apoptosis by recombinant human Trx [24]. Since I/R injury is a multi-factorial and results from oxidative protein modifications, mitochondrial dysfunction and alteration of redox state, we hypothesized that Trx would ameliorate I/R injury via regenerating oxidized proteins to their native state in the face of I/R –mediated modifications, and due to preservation of redox state in aged mice.

To determine specific redox-related mechanisms by which Trx ameliorates I/R injury in aging, we utilized *Trx-Tg* and *dnTrx-Tg* mice [25]. The *dnTrx-Tg* mice maintain only low amounts of active Trx (3-5 fold lower) because of a dominant-negative effect of the mutant protein in preventing redox-related actions of Trx via competitive inhibition for reduction by TrxR1 [25]. We show that overexpression of Trx in mice protects against I/R injury by preserving myocardial redox balance, protecting mitochondrial structure and function, improving mitochondrial biogenesis by maintaining PGC1α expression and rescue of ACO2, MFN1 and MFN2 expression in I/R via AKT-CREB pathway.

## RESULTS

### High levels of Trx in aged mice heart prevents I/R mediated redox shift

**Figure 1:**
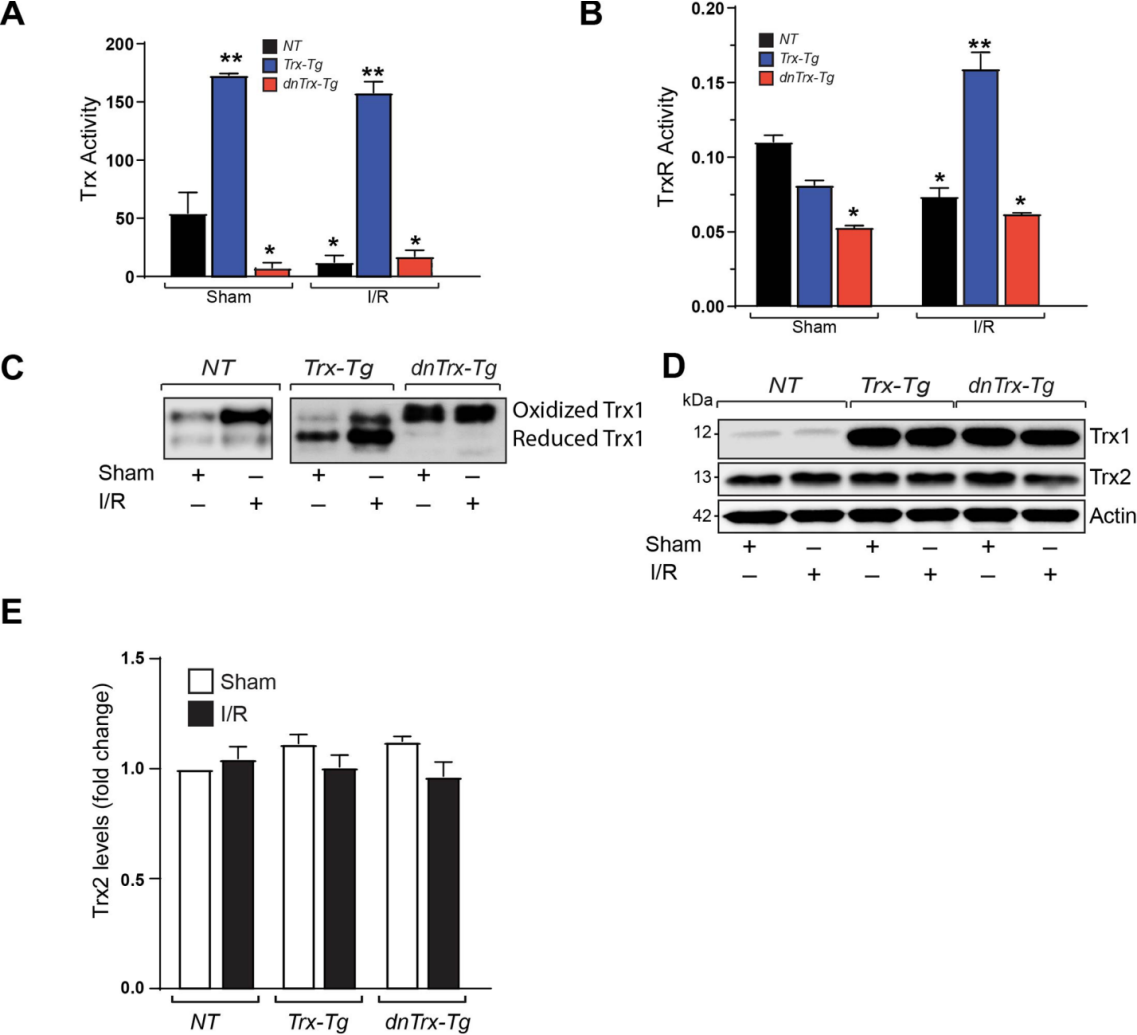
**High amounts of hTrx in transgenic mice prevents I/R mediated redox shift, and loss of Trx and Trx reductase activities.** (**A**) Trx activity was assayed in myocardium derived from sham and I/R-subjected *NT*, *Trx-Tg*, and *dnTrx-Tg* mice and expressed as nanomoles of NADPH oxidized per minute per milligram of protein at 25°C. Values are represented as means ± SEM (*n* =3-4). *p <0.05 versus NT sham; **p <0.05 versus *NT* or *dnTrx-Tg*. (**B**) TrxR activity in sham or I/R myocardium were expressed as micromoles of 5-thio-2-nitrobenzene (TNB) formed per minute per milligram of protein at 30°C. Values are represented as means ± SEM (*n* =3-4). *p <0.05 versus NT sham; **p <0.05 versus *NT* or *dnTrx-Tg* I/R. (**C**) Redox Western blot analysis revealing the redox state of Trx (oxidized and reduced) in sham or I/R myocardium from *NT*, *Trx-Tg* and *dnTrx-Tg* mice. (**D**) AAR region of sham or I/R myocardium from *NT*, *Trx-Tg* and *dnTrx-Tg* were lysed using M-PER lysis buffer and analyzed for Trx1, Trx2 and Actin by western blotting. (**E**) Trx2 levels were quantified and expressed as fold change. Statistical significance was determined with one-way ANOVA followed by Tukey’s post-hoc multiple comparisons test.

We determined the Trx redox state in aged mice heart and the effect of I/R to delineate how Trx may modulate myocardial redox in I/R. As shown in Figure 1A, 1B, the activities of Trx and TrxR1 are significantly lower in sham myocardium from *dnTrx-Tg* mice in contrast to those from *NT* or *Trx-Tg* mice, demonstrating that *dnTrx-Tg* mice have significantly decreased level of redox-active Trx and TrxR1 in the myocardium. Further, I/R caused marked reduction in Trx and TrxR1 activity in the infarcted region of *NT* mice (Figure 1A and B). However, infarcted myocardium from *Trx-Tg* mice showed higher Trx and TrxR1 activities compared to NT or *dnTrx-Tg* mice. I/R did not alter the Trx and TrxR activities further in *dnTrx-Tg* mice compared to respective sham animals (Figure 1A, 1B). We reasoned that oxidized Trx might have been accumulated in *dnTrx-Tg* mice heart in I/R due to oxidation of endogenous Trx. As shown in Figure 1C, Trx remained in oxidized state in sham or I/R treated *dnTrx-Tg* mice heart. However, Trx redox state in the infarcted myocardium from *Trx-Tg* mice was predominantly reduced, demonstrating that overexpression of Trx preserves overall redox state of the myocardium in I/R. Due to very low levels of endogenous Trx in mice, we ran separate western analysis (with higher amounts of protein) of aged sham *NT* or I/R subjected mice. As shown in Figure 1C (left panel), aged *NT-I/R* mice showed high level of oxidized Trx compared to sham treated mice. However, over expression of Trx or dnTrx did not affect Trx-2 levels (Figure 1D, 1E). Taken together, our data show that 3-fold higher active Trx in mice from the beginning of life preserves Trx redox in reduced state that protected against I/R -mediated Trx oxidation.

### High levels of Trx protect against I/R-mediated LV dysfunction, reduce infarct size and decrease the expression of apoptotic proteins in aged heart

**Figure 2:**
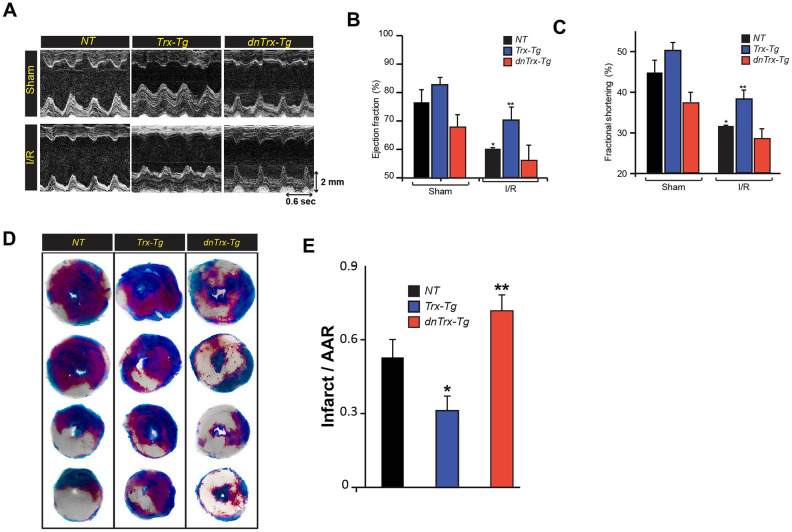
**High level of Trx protects against I/R mediated LV dysfunction and infarction of aged myocardium.** (**A**). Representative M-mode images taken from *NT*, *Trx-Tg* and *dnTrx-Tg* hearts of sham (top) and I/R (bottom). Ejection fraction; EF (**B**) and fractional shortening; FS (**C**), an index of cardiac contractile function, was determined by echocardiographic analysis. *p <0.05 versus *NT* sham; **p <0.05 versus *NT* or *dnTrx-Tg* I/R hearts, n=3-5. (**D**) *NT*, *Trx-Tg* and *dnTrx-Tg* mice were subjected to 60 min ischemia and 30 min reperfusion and then TTC staining was performed as described in the methods section. TTC stains viable tissue brick red and necrotic tissue as white. (**E**) Infarct area in relation to area-at-risk (AAR). *p <0.05 versus *NT* or *dnTrx-Tg* I/R; **p <0.05 versus *NT* or *Trx-Tg* I/R, n=4. Statistical significance was determined with the Student’s t-test.

We next evaluated the effect of Trx on cardiac function in I/R. We performed echocardiography on aged *NT*, *Trx-Tg* and *dnTrx-Tg* mice after 60 minutes of ischemia and 30 minutes of reperfusion. As shown in Figure 2A, 2B, I/R decreased the LV ejection fraction (EF) in *NT* mice compared to sham animals. In contrast, *Trx-Tg* mice were significantly protected from I/R -mediated reduction in EF compared to *NT* or *dnTrx-Tg* mice. *NT* and *dnTrx-Tg* mice also exhibited loss of fractional shortening (FS) during I/R compared to sham animals (Figure 2C). However, Trx-Tg mice showed significant improvement in EF compared to *NT* or *dnTrx-Tg* mice subjected to I/R (Figure 2C). Next, we determined the effect of high levels of Trx on infarct size by TTC staining. As shown in Figure 2D, 2E, aged *Trx-Tg* mice showed significant reduction of infarct size in I/R compared to *NT* mice. Lower levels of active Trx in *dnTrx-Tg* mice accentuated the I/R insult, as evidenced by significantly higher infarct size in *dnTrx-Tg* mice compared to *NT* or *Trx-Tg* (Figure 2E).

**Figure 3:**
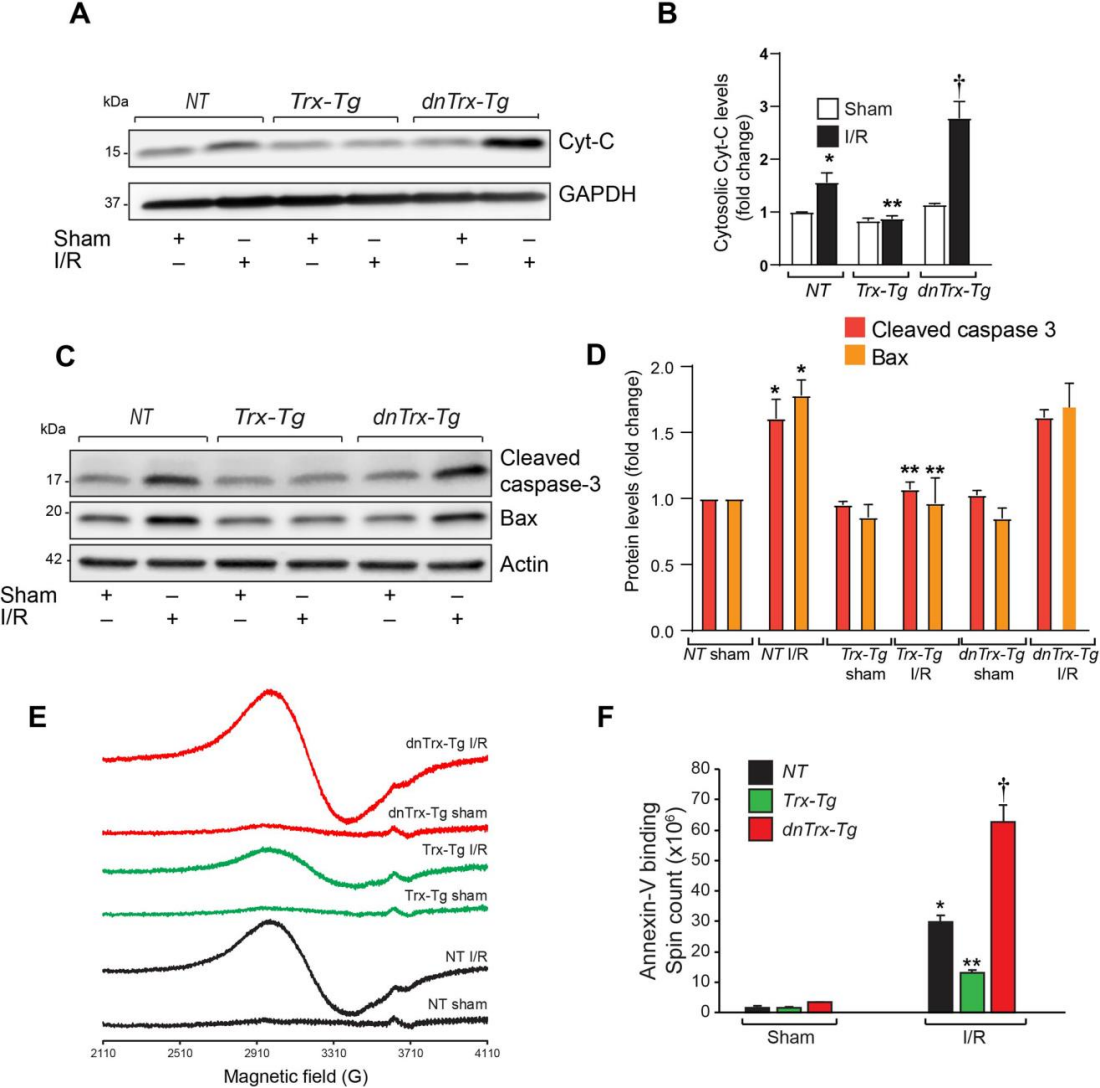
**High level of Trx protects against I/R- induced apoptosis.** (**A**) Cytosolic extract was prepared from AAR region of sham or I/R subjected *NT*, *Trx-Tg* and *dnTrx-Tg* mice and level of Cyt-C was analyzed by western blotting. (**B**) Levels of cytosolic Cyt-C was quantified and expressed as fold change. *p <0.05 versus *NT* sham; **p <0.05 versus *NT* or *dnTrx-Tg* I/R; † p <0.05 versus *NT* or *Trx-Tg* I/R. (**C**) AAR region of sham or I/R myocardium from *NT*, *Trx-Tg* and *dnTrx-Tg* were lysed using M-PER lysis buffer and analyzed for cleaved caspase 3 and Bax by western blotting. (**D**) Protein levels were quantified and expressed as fold change. *p <0.05 versus *NT* sham; **p <0.05 versus *NT* or *dnTrx-Tg* I/R hearts, n=3. (**E**) EPR spectra of paramagnetic iron bound Annexin-V. (**F**) Graph shows an absolute spin count of Fe-Annexin-V. Values are means ± SD (n = 3 mice). *, p < 0.01 versus *NT* Sham; **, p < 0.01 versus *NT* I/R or *dnTrx-Tg* I/R; † p <0.01 versus *NT* or *Trx-Tg* I/R. Statistical significance was determined with the Student’s t test (**B**, **D**) and one-way ANOVA followed by Tukey’s post-hoc multiple comparisons test (**F**).

Since apoptosis is a major contributing factor in I/R-induced death of myocytes resulting in MI, we analyzed the apoptotic markers such as release of cytochrome-c in cytosolic extracts from AAR region of sham or I/R subjected mice hearts. As shown in Figure 3A, 3B, I/R resulted in significant increase in cytochrome-c release in *NT* mice, however, the increase in cytochrome-c in *dnTrx-Tg* mice in I/R was 2-fold higher compared to *NT* mice. In contrast, there was no increase in cytochrome-c release in *Trx-Tg* mice subjected to I/R (Figure 3A, 3B). Next, we analyzed the level of cytochrome-c effector proteins, such as cleaved caspase -3 and proapoptotic Bax levels in sham or I/R to delineate the specific apoptotic pathway. As shown in Figure 3C, 3D, I/R resulted in elevated levels of cleaved caspase 3 and Bax. However, cleaved caspase levels were diminished in *Trx-Tg* mice in I/R. We performed an EPR apoptosis assay to determine the apoptosis in the entire infarcted region by removing the total heart tissue irrigated by LAD. As shown in Figure 3E, 3F, we found significant increase in iron-bound annexin-V in I/R in *NT* mice heart. In addition, the magnitude of apoptosis was further increased in *dnTrx-Tg* mice in I/R. *Trx-Tg* mice in I/R demonstrated significant decrease in myocardial apoptosis. Taken together, these data show that 3-fold increase in functional Trx in the aged myocardium protected against I/R -mediated LV dysfunction, decreased MI and reduced apoptosis. In contrast, loss of redox active Trx in the *dnTrx-Tg* mice exaggerated I/R injury with severe MI and apoptosis in the aged myocardium.

### High levels of Trx protect cardiac mitochondrial structure, function and prevents mitochondrial DNA damage due to I/R in aged mice

Since we found decreased levels of cytochrome-c, bax and cleaved caspase-3 in *Trx-Tg* mice in I/R, which are indicative of mitochondrial pathway of apoptosis, we reasoned that mitochondrial dysfunction might have been impacted by high levels of Trx in the hearts of *Trx-Tg* mice in I/R. Therefore, we determined the effect of high levels of Trx on mitochondrial function, mitochondrial ultrastructure, mitochondrial DNA damage and mitochondrial enzyme activities in sham and I/R animals. We analyzed the ultrastructure of myocardium by electron microscopy. As shown in Figure 4A, I/R caused significant loss of cristae in *NT* mice (*upper left panels*) and *dnTrx-Tg* mice in I/R (*lower left panels*). In contrast, the mitochondrial structure was preserved in *Trx-Tg* mice that showed normal cristae structure and density in I/R (*top right panels; and* Figure 4B). Additionally, as shown in Figure 4C, I/R caused significant increase in damaged mitochondria in aged *NT* or *dnTrx-Tg* mice, but not in aged *Trx-Tg* mice in I/R. Further, mitochondrial swelling was increased in I/R in aged *NT* or *dnTrx-Tg* mice, but not in aged *Trx-Tg* mice (Figure 4D).

**Figure 4:**
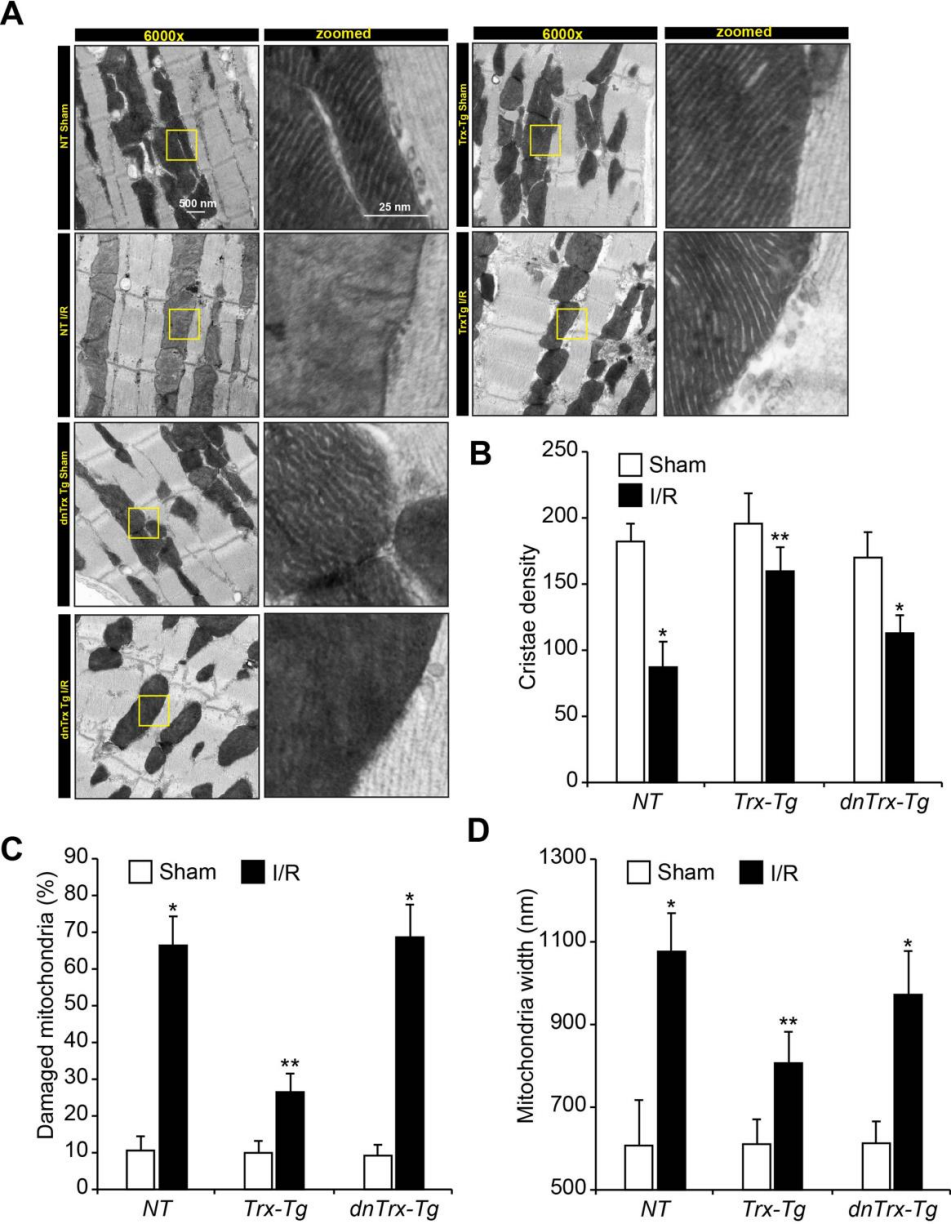
**High level of Trx prevents I/R-induced mitochondrial cristae and DNA damage.** (**A**) Ultrastructural analysis of sham or I/R hearts from *NT*, *Trx-Tg* and *dnTrx-Tg*. Representative transmission electron microscopic images showing cristae structure and density. Calculated cristae density (**B**), percent damaged mitochondria (loss of ≥50% cristae density) (**C**), and width of mitochondria (**D**) and plotted as bar graph. Values are means ± SD (n = 25). *, p < 0.01 versus Sham; **, p <0.01 versus *NT* or *Trx-Tg* IR.

**Figure 4:**
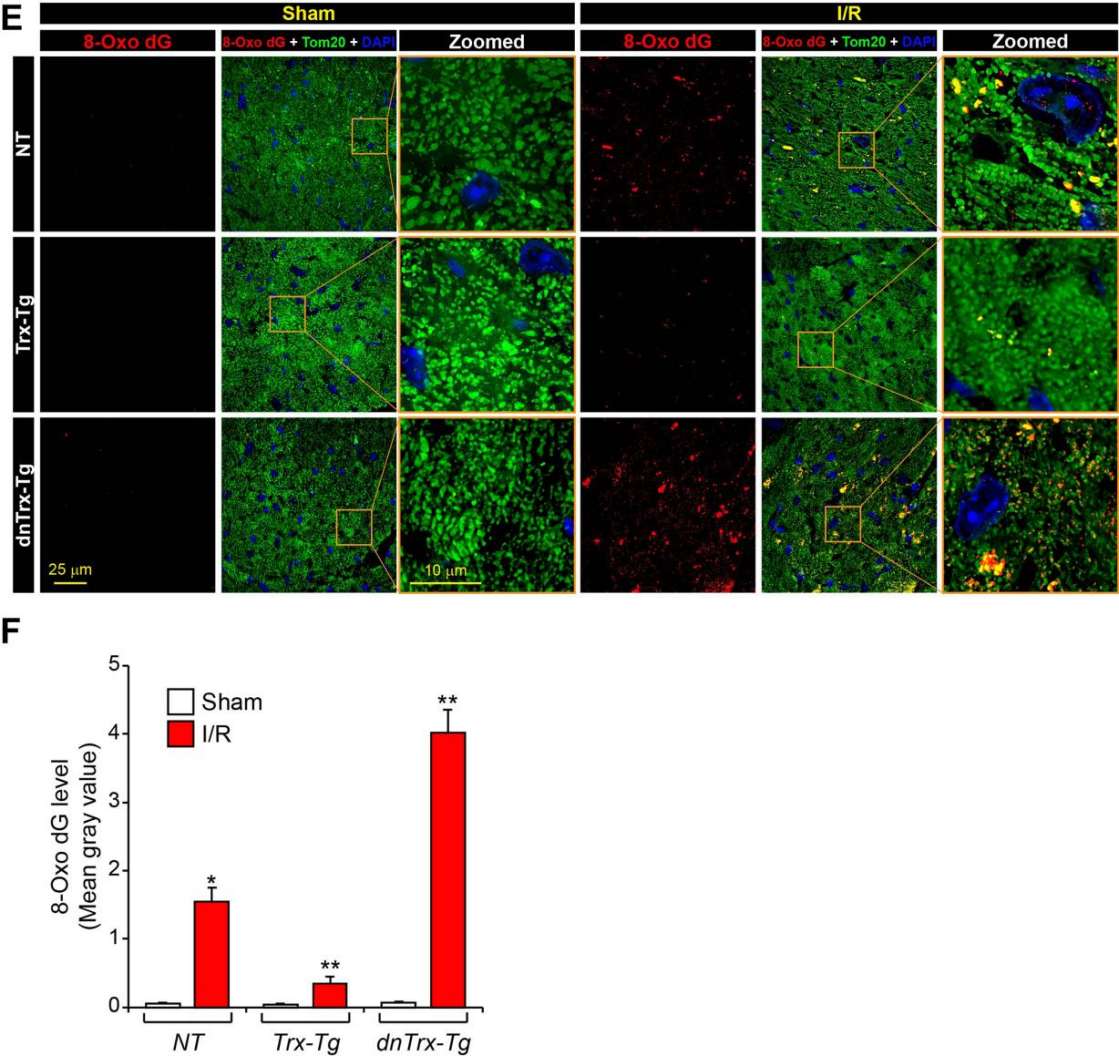
**High level of Trx prevents I/R-induced mitochondrial cristae and DNA damage.** (**E**) Immunofluorescence microscopic image shows accumulation of 8-oxo-dG in mitochondria of sham or I/R myocardium sections. (**F**) The levels of 8-oxo-dG was quantified and expressed as mean gray value. *p <0.05 versus NT sham; **p <0.05 versus *NT* or *dnTrx-Tg* I/R; † p <0.05 versus *NT* or *Trx-Tg* I/R. Statistical significance was determined with one-way ANOVA followed by Tukey’s post-hoc multiple comparisons test.

Mitochondrial genome encodes the core proteins of ETC [26], and mitochondrial DNA damage is known to occur in I/R. [27] Therefore, we sought to determine whether high levels of Trx would ameliorate mitochondrial DNA damage in I/R. As shown in Figure 4E, 4F (*top panels*), increased levels of 8-Oxo-dG were observed in the heart sections in *NT* and *dnTrx-Tg* mice in I/R (Figure 4E, 4F, *bottom panels*), indicating significant mitochondrial DNA damage. Additionally, *dnTrx-Tg* mice showed about 2.5 fold higher level of 8-Oxo-dG level compared to *NT* mice, demonstrating a critical role of Trx in preservation of mitochondrial DNA in I/R stress. In contrast, *Trx-Tg* mice hearts had significantly lower levels of 8-Oxo-dG in I/R (Figure 4E, 4F, *middle panels)*. These data demonstrate that Trx protects against mitochondrial DNA damage caused by I/R, which may protect mitochondrial ETC genes that, in turn, could preserve mitochondrial function in *Trx-Tg* mice in I/R.

**Figure 5:**
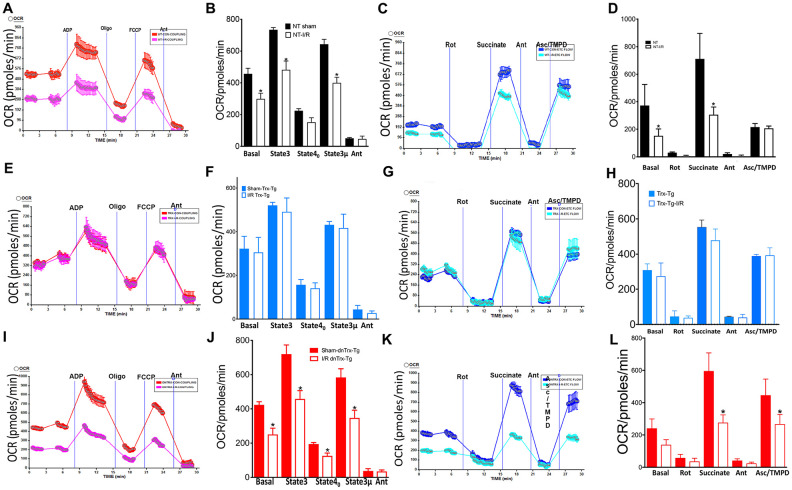
**Trx protects against I/R-induced mitochondrial dysfunction.** Effect of I/R on coupling and electron flow in mitochondria isolated from mice heart: Mitochondria were isolated from AAR region of sham or I/R subjected *NT*, *Trx-Tg* and *dnTrx-Tg* mice hearts as described in Materials and methods. (**A**) representative figure of XF24 coupling assay of sham or I/R mice mitochondria from (**A**) NT; (**E**) *Trx-Tg*; (**I**) *dn-Trx-Tg* mice, and OCR response to ADP, oligomycin, FCCP and antimycin A using point-to-point measurements. Graph of basal (state 2), state 3, and state 4_0_ respirations in sham and I/R mice heart mitochondria from (**B**) *NT*; (**F**) *Trx-Tg*; (**J**) *dn-TrxTg*. A representative OCR measurement figure of mitochondria from (**C**) *NT*; (**G**) *Trx-Tg*; (**K**) *dn-TrxTg* mice with pyruvate and malate as substrates, and the effect of rotenone, succinate, antimycin A and Asc/TMPD. Sham and I/R mouse mitochondria from (**D**) *NT*; (**H**) *Trx-Tg*; (**L**) *dn-TrxTg* mice with basal, rotenone, succinate, antimycin A and Asc/TMPD mediated OCR (sham = 3, I/R n = 3). Statistical significance was determined with one-way ANOVA followed by Tukey’s post-hoc multiple comparisons test. *P<0.05.

Since mitochondrial cristae harbor the ETC, which carryout oxidative phosphorylation for supply of energy to the heart, we speculated a protective role of Trx in maintaining the oxidative phosphorylation due to preservation of mitochondrial structure in I/R, as aged mice show mitochondrial dysfunction in I/R [28]. We evaluated mitochondrial function by assessing ADP stimulated respiration and pyruvate-malate –dependent electron flow in mitochondria isolated from hearts of sham or I/R subjected mice. As shown in Figure 5A, 5B, succinate-driven state 2 respiration was significantly decreased in *NT* animals in I/R, but not in *Trx-Tg* mice (Figure 5E, 5F). Additionally, ADP-coupled state 3 respiration was decreased in I/R in *NT* or *dnTrx-Tg* mice, although there was no significant difference in *Trx-Tg* mice (Figure 5A, 5B, 5I, 5J and Figure 5E, 5F). Oligomycin-mediated state 4 respiration in *NT* or *Trx-Tg* mice was similar in sham animals, but I/R -subjected *dnTrx-Tg* mice had significant decrease in state 4 compared to either *NT* or *Trx-Tg* mice, demonstrating significant proton leak in *dnTrx-Tg* mice in I/R [29]. Collectively, these data demonstrate that electron flow might have been impaired that promoted disturbed proton gradient resulting in differential oxygen consumption. Therefore, we analyzed the flow of electrons via mitochondrial ETC complexes. Complex-I-mediated respiration utilizing pyruvate and malate as substrates was decreased in *NT* or *dnTrx-Tg*, but not in *Trx-Tg* mice (Figure 5C, 5D, 5G, 5H, 5K, 5L). However, complex-II-driven respiration by succinate was significantly lower in *N*T and *dnTrx-Tg* mice, but not in *Trx-Tg* mice. Further, ascorbate-TMPD driven complex IV OCR was decreased only in *dnTrx-Tg* mice in I/R, but not in *NT* or *Trx-Tg* mice, demonstrating significant impact of Trx in electron flow via mitochondrial complexes in I/R. Taken together, these data demonstrate significant protective effect of high levels of Trx on mitochondrial structure, function and energy metabolism.

### Trx prevents I/R -induced loss of mitochondrial proteins, by upregulating transcription of PGC1α

**Figure 6:**
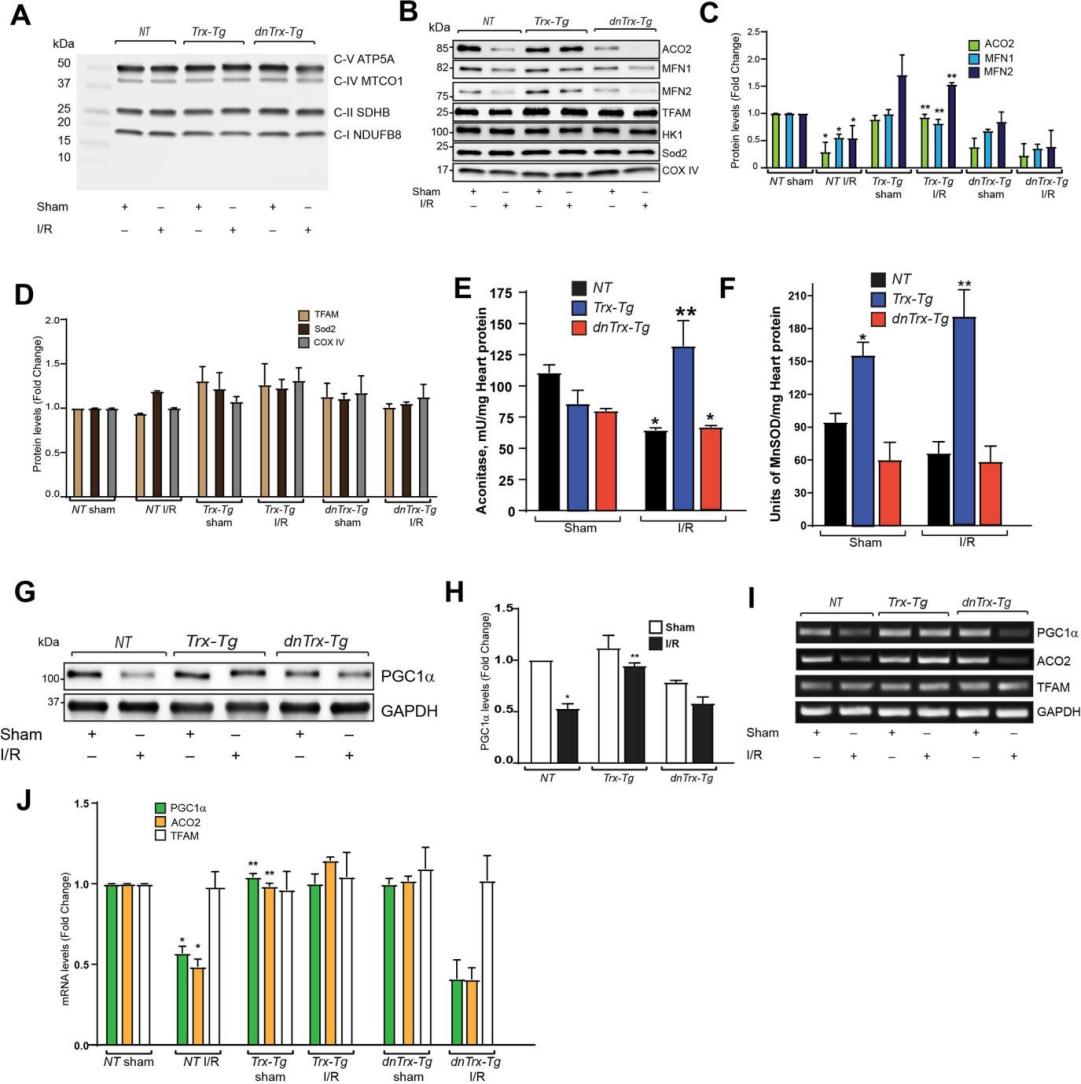
**Trx prevents I/R-induced loss of mitochondrial proteins, by upregulating transcription of PGC1α.** (**A**). Mitochondria was isolated from sham or I/R subjected *NT*, *Trx-Tg* and *dnTrx-Tg* mice. The mitochondrial extracts were analyzed for oxidative phosphorylation complex subunits by western blot using Abcam OXPHOS cocktail antibody. (**B**) Western blot analysis of ACO2, MFN1, MFN2, TFAM, Hexokinase 1 (HK-1), Sod2 and COX IV in sham or I/R mitochondrial extracts from *NT*, *Trx-Tg* and *dnTrx-Tg* mice. (**C**, **D**). Protein levels were quantified and expressed as fold change. *p <0.05 versus *NT* sham; **p <0.05 versus *NT* or *dnTrx-Tg* I/R. (**E**) Aconitase 2 activity and (**F**). MnSOD activity was determined in sham and I/R myocardium obtained from *NT*, *Trx-Tg*, or *dnTrx-Tg* mice as described in materials and methods. *p <0.05 versus respective sham; **p <0.05 versus *NT* or *dnTrx-Tg* I/R. n=3. (**G**) AAR region of sham or I/R myocardium from *NT*, *Trx-Tg* and *dnTrx-Tg* were lysed using M-PER lysis buffer and analyzed for PGC1α and GAPDH by western blotting. (**H**). Level of PGC1α was quantified and expressed as fold change. *p <0.05 versus *NT* sham; **p <0.05 versus *NT* or *dnTrx-Tg* I/R. (**I**). RT-PCR analysis of PGC1α, ACO2 and TFAM in sham and I/R myocardium (**J**). mRNA levels of PGC1a, ACO2 and TFAM were quantified and expressed as fold change. *p <0.05 versus *NT* sham; **p <0.05 versus *NT* or *dnTrx-Tg* I/R. Statistical significance was determined with the Student’s t test (**C**, **D**, **H**, and **J**) and one-way ANOVA followed by Tukey’s post-hoc multiple comparisons test (**E**, and **F**).

PGC1α is the master regulator of mitochondrial biogenesis and regulates several mitochondrial gene transcriptions as a coactivator of PPARγ. We analyzed the expression of mitochondrial proteins in mitochondrial extract from sham or I/R subjected mouse hearts. As shown Figure 6A, I/R did not change the expression of mitochondrial complexes, as analyzed by western blot using OXPHOS cocktail antibody (Abcam), which detects CI subunit NADH:ubiquinone oxidoreductase subunit8 (NDUFB8), complex II subunit 30kDa (CII-30kDa), CIII-Core protein 2, CIV subunit I and CV alpha subunit. We also analyzed aconitase (ACO2), mitofusin-1(MFN1), mitofusin-2(MFN2), Transcription factor A mitochondrial (TFAM), Hexokinase 1 (HK-1), superoxide dismutase-2 (Sod2) and cytochrome oxidase IV (COX IV) in mitochondrial extracts. As shown in Figure 6B, 6C, I/R resulted in significant loss of ACO2, MFN1 and MFN2 in *NT* or *dnTrx-Tg* mice in I/R mitochondrial extracts. However, high levels of Trx prevented I/R-induced loss of ACO2, MFN1 and MFN2 proteins (Figure 6B, 6C). I/R did not change the level of TFAM, HK-1, Sod2 and COX IV in NT, *Trx-Tg*, or *dnTrx-Tg* mice (Figure 6B, 6D).

Since ACO2 protein expression was decreased in I/R, we sought to determine the activity of aconitase in mitochondrial extracts. As shown in Figure 6E, I/R resulted in loss of aconitase activity in *NT* and *dnTrx-* *Tg* mice, but not in *Trx-Tg* mice, which had significantly higher aconitase activity. We also evaluated Sod2 (MnSOD) activity in I/R, as it is an important enzyme of mitochondrial matrix that dismutates O_2_^.-^ to H_2_O_2,_ and thereby decreases mitochondrial oxidative stress. Although I/R did not alter the MnSOD activity in *NT* or *dnTrx-Tg* mice, *Trx-Tg* mice had significantly higher activity in sham or I/R subjected mice (Figure 6F). ACO2 activity was strongly correlated with its higher expression in *Trx-Tg* mice and decreased expression in *NT* or *dnTrx-Tg* mice in I/R. We determined whether the expression of PGC1α is modulated in I/R. As shown in Figure 6G, 6H, I/R caused significant loss of PGC1α expression in hearts of *NT* mice. In contrast, *Trx-Tg* mice were protected against I/R induced loss of PGC1α. Further, as shown in Figure 6I, 6J, I/R decreased mRNA expression of PGC1α and ACO2, but not TFAM in *NT* or *dnTrx-Tg* mice. In contrast, *Trx-Tg* mice had significantly higher level of PGC1α and ACO2. These data indicate that expression of PGC1α is essential for Trx-mediated protection of mitochondrial function.

### Trx regulates expression of PGC1α via PI3K-AKT-CREB axis in cardiomyocytes

**Figure 7:**
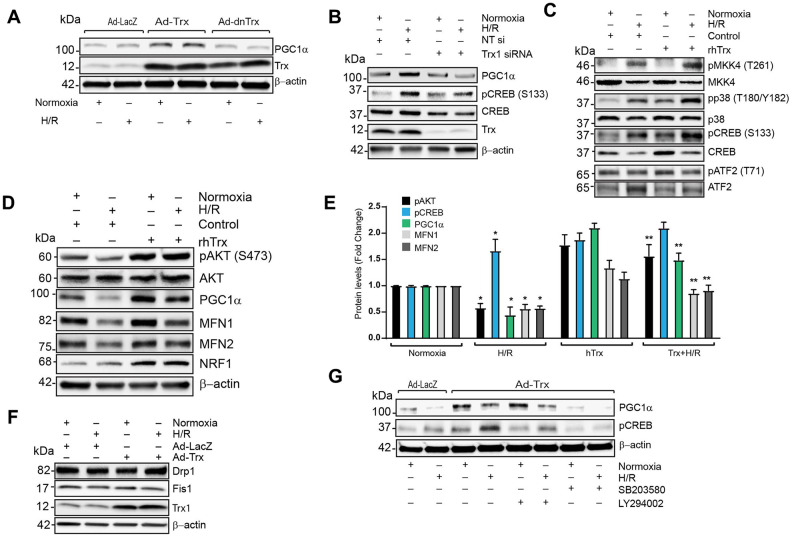
**Trx regulates expression of PGC1α via PI3K-AKT-CREB axis in cardiomyocytes.** (**A**). Western blot analysis of PGC1α, Trx and β-actin in lysate from Ad-LacZ, Ad-Trx and Ad-dnTrx infected and H/R (24/2h) exposed HCAECs. (**B**). Western blot analysis of PGC1α, pCREB (S133), CREB, Trx and b-actin in lysates from NT or Trx siRNA transfected and H/R (24/2h) exposed HCAECs. (**C** and **D**). Western blot analysis of pMKK4 (T261), MKK4, pp38 (T180/Y182), p38, pCREB (S133), CREB, pATF2 (T71), ATF2, pAKT (S473), AKT, PGC1α, MFN1, MFN2, NRF1 and β-actin in lysates from rhTrx pretreated and H/R (24/2h) exposed H9C2 cells. (**E**). Levels of protein in H9C2 cells were quantified and expressed as fold change. *p <0.05 versus Normoxia; **p <0.05 versus H/R. (**F**). Western blot analysis of Drp1, Fis1, Trx and β-actin in lysate from Ad-LacZ and Ad-Trx infected and H/R (24/2h) exposed H9C2 cells. (**G**). Western blot analysis of PGC1a, pCREB (S133) and β-actin in lysates from p38 inhibitor, SB203580 (2.5 μM) and PI3 kinase inhibitor, LY294002 (5.0 μM) pretreated and H/R (24/2h) exposed H9C2 cells. Statistical significance was determined with the Student’s t test.

PGC1α expression is regulated by CREB, MEF-2 and ATF2 transcription factors [30]. Therefore, we determined whether expression of PGC1α and its upstream pathways are modulated by Trx redox state. As shown in Figure 7A, overexpression of Trx increased PGC1α levels in human coronary artery endothelial cells (HCAECs), but not in cells with redox-inactive Trx expression, indicating Trx-dependent redox regulation is critically important for PGC1α expression. Next, we determined the effect of Trx deficiency on PGC1α expression and CREB phosphorylation. Trx depletion inhibited hypoxia/reoxygenation (H/R) -induced expression of PGC1α and phosphorylation of CREB (Figure 7B). However, H/R did not downregulate the expression of PGC1α as observed in I/R (Figure 7B). Since major cell type in the heart is composed of endothelial cells, cardiomyocyte and fibroblast [31], we performed further studies with neonatal cardiomyocytes H9C2 to evaluated PGC1α regulation in cardiomyocytes. We have previously shown that MKK4 activation is regulated by Trx redox state [19]. Additionally, Trx activates MKK4 and PI3 kinase pathways and these signaling cascades are known to regulate PGC1α transcription via activation of CREB or ATF2 [19, 23]. As shown in Figure 7C, 7D, treatment of H9C2 with recombinant human Trx (rhTrx) activated MKK4, p38 and AKT. Pretreatment of rhTrx prevented H/R-induced loss of pAKT, PGC1α, MFN1 and MFN2, but potentiated the activation of MKK4, p38 and CREB (Figure 7C–7E). We also found that higher level of NRF1 transcription factor in rhTrx pretreated samples and there was no change in pATF2 level (Figure 7C, 7D). Since rescue of mitochondrial fusion proteins MFN1 and MFN2 was observed in rhTrx pretreated H9C2 cells exposed to H/R, we analyzed effect of Trx on proteins involved in mitochondrial fission, such as Drp1 or Fis1. As shown in Figure 7F, there was no change in the expression of Drp1 or Fis1 in H/R in presence or absence of Trx. Although our data demonstrate that both p38 and AKT are activated by rhTrx, we sought to delineate the specific pathway that activates Trx-mediated PGC1α expression in H/R. As shown in Figure 7G, inhibition of PI3 kinase blocked the Ad-Trx mediated expression of PGC1α and CREB activation, but not p38. Collectively, our data show that Trx regulates the expression of PGC1α via activation of PI3K-AKT-CREB axis. Further, elevated level of PGC1α in Trx treated cells or Trx-Tg mice upregulates the expression of ACO2, MFN1 and MFN2 by coactivating their transcription factors.

## DISCUSSION

Aging is an independent risk factor for cardiovascular disorders including ischemic heart diseases [32]. A decline in cardiac mitochondrial function is a major contributor to age-related decrease in tolerance of the heart to stress such as I/R [6]. Continuous oxidative events over the life-span due to oxygen metabolism modifies mitochondrial proteins, impairing their critical role in energy production, and to overcome further oxidative insult such as I/R. Although ROS are unequivocally implicated in mitochondrial dysfunction in ischemic heart diseases such as I/R [4], antioxidant interventions have not only provided inconclusive results, but also failed to find their way into clinical translation. Accumulating evidence suggests that the mechanism of reperfusion injury such as myocardial infarction is complex and multi-factorial [33]. Oxidative protein modifications and mitochondrial dysfunction due to advancing age, compounded with I/R insult exert severe damage to the myocardium in aged individuals in I/R. Therefore, it is likely that one specific antioxidant might be ineffective to a multi-factorial damage to the aging heart in I/R injury.

The present study was undertaken to evaluate role of Trx in I/R injury in aged mice, as Trx is a unique multifunctional redox protein that regenerates oxidatively inactivated protein [17, 34], scavenges deleterious ROS such as hydroxyl radicals and singlet oxygen, induces mitochondrial superoxide dismutase-2 (SOD2) [18], and modulates various signal transduction pathways. We found that overexpression of Trx (2.5 to 3-fold) since the beginning of life prevents I/R injury in aged transgenic mice, as evidenced by reduced infarct size and decreased myocardial apoptosis. Trx improved left ventricular function by protecting against I/R -induced reduction in EF and FS. Additionally, high levels of Trx in *Trx-Tg* mice rescued structural and functional impairment of mitochondria and improved mitochondrial energy metabolism in I/R. Using Trx-deficient mice (*dnTrx-Tg*), we found that the decreased levels of active Trx (about 4-fold decrease in activity) exacerbate I/R-induced infarct size and myocardial apoptosis. Further, Trx prevented I/R-induced loss of PGC1α mRNA and protein expression, resulting in protection of I/R-mediated decreased expression of ACO2, MFN1, and MFN2. Further, Trx-mediated activation of AKT-CREB-PGC1α signaling cascade is essential for the regulation of mitochondrial gene expression and function in I/R.

Three-fold higher expression of Trx in mice prevented oxidation of Trx in I/R, and showed increased TrxR1 activity, preserving the redox state of myocardium during I/R insult in contrast to mice with decreased levels of active Trx. This is important for further protective role of Trx as Trx itself undergoes oxidation and other modifications. Oxidative modifications of mitochondrial proteins, such as carbonylation, S-glutathionylation, and accumulation of protein disulfides and mixed disulfides in the aged myocardium alter the structure and function of mitochondria during the aging process. [35] These modifications lead to decline in mitochondrial respiration, mitochondrial content, and the accumulation of defective mitochondria resulting in ROS production, oxidative injury, and cell death [6]. Aging heart with these incapacitated mitochondria acutely fails to overcome or adapt to I/R insult resulting in life-threatening myocardial infarction [9]. We have previously shown that mitochondrial succinate-dependent energy coupling with addition of ADP significantly declines in aged mice [6]. Additionally, the electron transfer due to oxidation of pyruvate/malate via complex I to IV significantly decreases in aged mice [6]. Further, loss of aconitase expression and activity in I/R in *NT* or *dnTrx-Tg* mice, but not in *Trx-Tg* mice potentially decrease NADH production and loss of electron flow. A previous study has shown decreased aconitase activity without decrease expression in rat I/R model, where the entire heart tissue was evaluated after I/R in *ex vivo* Langendorff isolated heart model with retrograde perfusion. [36] In contrast, in our study, we evaluated mitochondrial function *in vivo* and found that decreased aconitase expression in the infarcted area in I/R. Additionally, studies have demonstrated that α-ketoglutarate dehydrogenase (αKGDH) and aconitase of TCA cycle are inactivated in I/R due to oxidation of critical sulfhydryl and inactivation of [4Fe-4S]^2+^ cluster center in aconitase [37]. Since Trx reduces oxidized proteins, and we did not observe decreased aconitase activity or decreased electron flow in Trx-Tg mice, it is likely that high levels of Trx might have reversed aconitase and αKGDH to their native functional state due to its disulfide reducing properties. We found that mitochondrial coupling and electron transfer via complex I to complex IV remains functional in the mitochondria isolated from *Trx-Tg* mice in I/R, but not from *NT* or *dnTrx-Tg* mice, which is correlated with protection of aconitase expression and activity in *Trx-Tg* mice, indicating a redox-related mechanism. In this regard, aconitase activity was restored in aged *Trx-Tg* mice in I/R, but not in *dnTrx-Tg* mice. Further, the activity of Sod2 was increased in *Trx-Tg* mice in I/R in contrast to *dnTrx-Tg* mice, indicating loss of antioxidative function due to lack of redox-active Trx.

Enhancing mitochondrial fusion or preventing the mitochondrial fission has been shown to protect against I/R injury [10]. Conditional knock out of Mfn1 and Mfn2 in adult hearts induced mitochondrial fragmentation, mitochondrial respiratory dysfunction, which lead to dilated cardiomyopathy [38]. Additionally, previous studies have shown that loss of mitofusin during I/R constitutes a mechanism of I/R injury. [39] In the present study, we demonstrated that Trx prevented the loss of MFN1 and 2, which could be another mechanism related to Trx-mediated protection of mitochondrial dysfunction in I/R. Collectively our data show that 2.5 to 3-fold increase in Trx prevents loss of expression of MFNs and aconitase and thus preserved the mitochondrial structure and function during I/R.

PGC1α regulates the expression of several mitochondrial genes via its binding to transcription factors such as ERRs, PPARs, and NRFs [13]. The PGC1α KO mouse shows decreased expression of mitochondrial oxidative phosphorylation genes, mitochondrial enzymatic activities and reduced levels of ATP [40]. In the present study, we found decreased PGC1α mRNA and protein expression in I/R, which was restored by Trx overexpression. Consistent with this finding an earlier study has shown upregulation of PGC1α by Trx in mouse heart [41]. Although unclear, we speculate that this finding could be due to the cardiac-specific expression of PGC1 α and the use of the *ex vivo* Langendorff global ischemia model. In our *in vivo* and cell culture studies we found that PGC1α expression was inhibited by I/R or H/R, respectively. Since PGC1α transcription is regulated by CREB [30], ATF2 [42], and MEF2 [43] transcription factors, we found that Trx upregulates PGC1α expression by activation of the AKT-CREB signaling. In a previous study we have demonstrated the activation of AKT by Trx [23].

In conclusion, our data established that high levels of Trx preserved Trx redox state and mitochondrial structure, resulting in mitochondrial integrity in I/R. Additionally, Trx rescued aconitase and Sod2 activity via its disulfide reductase properties that allowed uninterrupted mitochondrial energy production during I/R via enhanced coupling and flow of electrons, and substrate oxidation via functional mitochondria. High levels of Trx also promoted mitochondrial biogenesis by promoting the expression of PGC1α via PI3K-AKT-CREB pathway during I/R. Upregulation of P13K-AKT-CREB- PGC1α axis prevents the loss of MFN1, MFN2, ACO2 and also maintained the activity of aconitase resulting in improved mitochondrial function and decreased apoptosis.

## MATERIALS AND METHODS

### Antibodies and chemicals

Antibodies and chemicals were obtained from following vendors. Abcam (Cambridge, MA): Total OXPHOS Rodent WB Antibody Cocktail (ab110413), anti-aconitase 2 (110320), anti-Hexokinase 1 (ab150423) and anti-TOMM20 (ab205486); BD Bioscience: Anti-Drp1 (611738) and anti-Cytochrome C (556433); Cell Signaling Technologies (Danvers, MA): Anti-Trx (2298), anti-Cleaved caspase-3 (9661), anti-Bax (2772), anti- Phospho-CREB (Ser133) (9191), anti-Phospho-Akt (Ser473) (4060), anti-AKT (9272), anti-Phospho-SEK1/MKK4 (Thr261) (9151), anti-SEK1/MKK4 (9152), anti-Phospho-p38 MAPK (Thr180/Tyr182) (4511), anti p38 MAPK Antibody (9212) and anti-Phospho-ATF-2 (Thr71) Antibody (9221); Santa Cruz Biotechnology (Dallas, TX): Anti-Actin (sc-1616), anti-Mfn1 Antibody (sc-50330), anti-COX4 (sc-58348), anti-NRF-1 Antibody (sc-33771), anti-ATF-2 Antibody (sc-187) and anti-Fis1 Antibody (sc-98900); Novus Biologicals (Centennial, CO): anti- PGC1 alpha Antibody (NBP1-04676); Sigma (St. Louis, MO): Anti-MFN2 antibody (WH0009927M3), Anti-MnSOD Antibody (06-984), Anti-8-Oxoguanine Antibody (MAB3560), anti-TFAM, anti-GAPDH antibody-HRP conjugate, anti β-actin-HRP conjugate, and recombinant human Trx; Thermo Scientific (Waltham, MA): Alexa Fluor 488, 568, 647-conjugated secondary antibodies, secondary anti-rabbit, anti-mouse IgG-HRP antibodies, isolectin IB4-Alexa Fluor 568 and M-PER Mammalian Protein Extraction Reagent; All other chemicals were purchased from Sigma unless otherwise stated.

### Animals and cells

Wild-type C57BL6 strain (*WT*) were purchased from Charles River Laboratory. Transgenic mice with overexpression of human Trx (*Trx-Tg*) or dominant-negative Trx (*dnTrx-Tg*) were bred and maintained in the animal facility of Texas Tech University Health Sciences Center and have been described previously [25]. Both males and females were used in this study. All mice strains used in this study are from a C57BL/6 background and are 20-26 months of age, equivalent to human age of 70-75 years [44]. All animal procedures were approved by the Institutional Animal Care and Use Committee (IACUC) of the Texas Tech University Health Sciences Center and were consistent with the Guide for the Care and Use of Laboratory Animals published by the National Institute of Health. HCAEC were purchased from Clonetics and propagated in endothelial basal medium supplemented with additives (Bullet kit, Clonetics). H9c2 cells were purchased from ATCC and propagated in DMEM with 10% FBS.

### Cell culture and hypoxia/ reoxygenation (H/R)

HCAECs and H9C2 cells in complete medium were flushed with a 95% N2, 5% CO_2_ gas mixture while in a Billups-Rothenberg modular chamber to create a hypoxic environment. The oxygen level was kept below 1% by measuring with an oxygen electrode. Chambers were kept inside the incubator at 37 °C for indicated periods of time and followed by 2 h of reoxygenation in normoxic condition.

### Myocardial ischemia and reperfusion

*NT*, *Trx-Tg* and *dnTrx-Tg* littermates were anesthetized with ketamine (100 mg/kg) and xylazine (10 mg/kg), by injecting via intra peritoneal route (i.p.). After an equilibration period of 10 min, the left thoracotomy was performed in the fourth intercostal space, and the pericardium was opened to expose the heart. An 8-0 silk suture was passed around the LAD at a point two-thirds of the way between its origin near the pulmonary conus and the cardiac apex. Coronary artery occlusion was achieved by ligating the left descending coronary artery using a slipknot. Following 60 minutes of ischemia, the slipknot was released, and the myocardium was reperfused for 30 minutes. Sham mice underwent the same procedure without the slipknot tied. Mice were sacrificed after 60 minutes of ischemia followed by 30 minutes of reperfusion. To collect heart samples, mice were euthanized by injecting ketamine (100 mg/kg) and xylazine (10 mg/kg) via i.p route.

### Adenovirus production

AdenoX system was obtained from Stratagene Corp. (La Jolla, CA), and LacZ or Trx cDNA was cloned into pAdenoX vector as described previously [45]. Recombinant virus was allowed to infect HEK293 cells for generation of viral particles.

### Determination of infarct size

Myocardial infarct size was determined as described previously [46]. Briefly, after reperfusion, animals were sacrificed, and the aortae were cannulated and perfused with saline to remove blood. 0.25 ml of 1.5% Evans blue was perfused after religating the coronary artery to demarcate remote myocardium (blue) and AAR. 1.0-mm heart sections were made using mouse coronal matrix and stained with 1.0% triphenyltetrazolium chloride (TTC) for 15 min at 37 °C. After TTC staining, paraformaldehyde-fixed heart sections were photographed using Nikon D5200 camera using Nikon AF-S DX NIKKOR 18-55 mm lens at f/6.3, 1/160s, ISO200. TTC stained and unstained area (infarct) at AAR was quantified using Adobe Photoshop.

### Myocardial echocardiography

Transthoracic echocardiography was performed on anesthetized mice using a Visual Sonics Vevo3100 (Toronto, ON, Canada) Imaging System with a 30-MHz high-frequency transducer (MX400). After sham or I/R surgery, mouse under Ketamine and Xylazine anesthesia was laid supine on a heated platform, and M-mode images were recorded at the level of the papillary muscle. The left ventricular ejection fraction (EF%) and fractional shortening (FS %) were calculated using Vevo LAB software.

### Electron microscopy

Sham and I/R subjected hearts were immediately fixed by a retrograde perfusion with 2% glutaraldehyde in PBS. Then, 1.0 mm thick sections were prepared from AAR region and stored in 4% glutaraldehyde in PBS. Ultrathin sectioning was completed in Electron Microscopy Core Facility, UT Southwestern Medical Center, Dallas, TX. Ultrastructure images from copper grid mounted ultrathin section of samples were obtained using Hitachi High-Technologies H-7650 Transmission electron microscope in College of Arts and Sciences Microscopy, Texas Tech University, Lubbock, TX. Mitochondrial cristae density was calculated from the inverse of calibrated mean gray value from the electron-dense area inside the inner-mitochondrial membrane of intrafibrillar mitochondria. Total and damaged mitochondria numbers were counted from 6,000x magnification TEM images, and percent damaged mitochondria was calculated. Mitochondria with loss of ≥50% cristae density were considered damaged mitochondria. Mitochondria width was calculated from 10,000x TEM images.

### RNA interference

Small interfering RNAs were obtained for nontargeting siRNA control, and hTrx from Dharmacon Inc. (Arvada, CO). 100 nM of siRNA was transfected using lipofectamine RNAiMAX reagent obtained from Thermo Scientific (Waltham, MA). Inhibition of gene expression by siRNA was determined after 36-48 h by Western blotting.

### RT-PCR

Total RNA was isolated from RNA*later* preserved AAR region of mouse hearts using TRIzol (Cat. No. 15596-018, Ambion), and cDNA was generated by reverse transcription reaction using high-capacity cDNA Reverse Transcription Kit (Cat. No. 4374966, Applied Biosystems). The cDNA was then used as a template for PCR amplification using the following primers:

### Western blotting

Protein extracts were prepared from appropriately treated H9C2, HCAEC cells or sham or infarcted left ventricle using M-PER mammalian protein extraction reagent from Thermo Scientific (Waltham, MA) with protease and phosphatase inhibitors. The homogenates were centrifuged at 10, 000 rpm for 10 min at 4°C. Protein concentrations were determined with the BCA protein assay kit (Pierce Chemical, Rockford, IL). For analysis of Cyt-C, cytosolic and mitochondrial extracts were prepared using Abcam Mitochondria Isolation Kit for Tissue (ab110168). Protein extracts were analyzed by Western blotting using their specific antibodies.

**Table:** 

**Gene**	**Forward Primer**	**Reverse Primer**
mACO2	GGTGGCTGTACCATCAACCA	TTCACACCGATCACCTTGGG
mPGC1α	TTGGTGACCATGACTACTGT	AAGTCTCTCTCAGGTAGCAC
mTFAM	GTCACGAAGCTCAGTAGGCA	CTCCACAGGGCTGCAATTTT
mGAPDH	CCAGAACATCATCCCTGCAT	CATCGAAGGTGGAAGAGTGG

The amplified PCR products were separated on 2% agarose gels and stained with ethidium bromide. Gel images were captured using LI-COR odyssey Fc Imaging System.

### Trx redox state assay

Carboxymethylation of mice heart tissue was performed as described in our previous publications [15, 25]. Briefly, AAR region of heart tissue (10–20 mg) was homogenized in 0.5 ml carboxymethylation buffer (0.1 M Tris·HCl pH 8.8, 6 M guanidine hydrochloride, and 10 mg/ml iodoacetic acid), and after the addition of 5 μl of 10% Triton X-100 the samples were incubated for 1 h at 37°C in the dark. Samples were centrifuged in a tabletop refrigerated centrifuge (Eppendorf) for 10 min at 2,500 rpm, and the supernatants (0.5 ml) were transferred to desalting columns to remove guanidine hydrochloride. Protein content was determined by the Bradford method (Bio-Rad, Hercules, CA). Twenty micrograms of carboxymethylated heart tissue homogenate were fractionated on a 15% native polyacrylamide gel (Bio-Rad). Transferred to nitrocellulose membrane and probed with anti-Trx antibody.

### Immunofluorescence microscopy

Sham or I/R heart tissue sections were deparaffinized, hydrated, permeabilized, blocked, and immunostained with anti-8-Oxo-dG and anti-Tom 20 antibodies followed by Alexa Fluor 488- and Alexa Fluor 568-conjugated donkey anti-rabbit and anti-mouse secondary antibodies. Nuclei were counterstained with DAPI. Fluorescence images were obtained via 100x/1.4 NA objective using Zeiss Axio Imager Z2 upright fluorescent microscope. The fluorescence intensity was quantitated using Adobe Photoshop.

### Mitochondria isolation for XF24 assay

AAR region of sham or I/R hearts were surgically removed. Mitochondria from hearts of mice were isolated as described earlier [7]. 80 mg of AAR region of heart was minced and homogenized at 4 °C using Kimble Chase 2mL tissue grinder tube with sequential use of pestle A and pestle B in mitochondrial isolation buffer (70 mM sucrose, 210 mM mannitol, 5 mM HEPES, pH 7.2, 1 mM EGTA and 0.5% fatty acid free BSA). The homogenate was centrifuged at 5,000 ×*g* for 10 min. The supernatant was again centrifuged at 1600 *g* for 5 min. The supernatant was centrifuged at 12500g for 10 min at 4°C in Avanti J-E centrifuge using JA20 rotor. The translucent white pellet was resuspended gently in buffer A and centrifuged again in Avanti J-E centrifuge at 25000g for 5 min at 4°C. The mitochondrial pellet was suspended in mitochondrial isolation buffer without BSA and protein was estimated with Bradford assay (Biorad, Rockford, IL). Mitochondria were suspended at 1.5 μg/50 μl in 1× mitochondrial assay buffer (MAS; 70 mM sucrose, 220 mM mannitol, 10 mM KH_2_PO_4_, 5 mM MgCl_2_, 2 mM HEPES, 1 mM EGTA, and 0.2% fatty acid free BSA; pH 7.2 at room temperature) and plated into each well of the v7 assay plate of XF24 analyzer.

### XF24 instrument setup and analysis

Analysis of mitochondrial function was performed in XF24 flux analyzer (Seahorse, Bellerica, MA) as previously published. [7] Briefly, XF24 instrument was equilibrated at 37°C overnight. 1.5μg of mouse heart mitochondria was plated in each well of the XF24 v7 plate in a volume of 50 μl containing 1× MAS with 10 mM succinate and 2 μM rotenone as substrate for coupling assay; and 10 mM pyruvate, 2 mM malate and 4 μM FCCP was added to 1× MAS for the electron flow experiment as described in our previous publication [7].

### Thioredoxin and thioredoxin reductase assays

Trx and Trx reductase activity assay were performed in sham or myocardium as described in our previous publication [47]. All assays were performed in Beckman DU800 spectrometer with temperature control and using quartz cuvettes.

### Aconitase and SOD2 assays

Aconitase activity was measured in isolated mitochondria as described before [48] and SOD2 activity as described in our previous publication [7].

### Quantification of apoptosis in heart by EPR

To quantify total apoptosis in the infarcted tissue of mouse heart, we modified an assay method originally developed by Fabisiak et al. [49], using annexin-V magnetic microbeads kit from Miltenyi Biotec GmbH, Germany (Cat. No. 130-090-201). After sham or IR surgery, the heart was quickly isolated from mice, cannulated via aortic arch and perfused with ice cold saline followed by 1x annexin-V binding buffer supplied by the manufacturer. Following complete removal of circulating blood, the heart was perfused with 250 μL of annexin-V microbead suspension and incubated at 2-4°C for 20 minutes. At the end of the incubation period, the heart was perfused with ice cold 1x annexin-V binding buffer and the entire infarcted tissue (area lower to the occlusion site in the LAD) was dissected out. Total annexin-V bound to infarcted tissue was quantified by measuring conjugated iron spins using Bruker EMX Micro spectrometer at room temperature. EPR spectra were acquired under following scan conditions: microwave frequency, 9.83 GHz; power, 30 mW; attenuation 8 dB; modulation frequency, 100 kHz; modulation amplitude, 4.00 G; sweep time, 60 s; time constant, 20.48 s; receiver gain, 20 dB; magnetic field, 2110-4110 G. Absolute spin counts from spectra were calculated using Quantitative EPR module of Bruker Xenon Micro 1.3 software.

### Statistical analysis

The experiments were performed in triplicate and repeated for a minimum of 2 times. All cell culture studies were performed in triplicate and repeated at least twice. Data were statistically analyzed by analysis of variance for multiple means with Tukey's post hoc analysis. Student's t-test was used to compare two means. Prism software (Version 8.0) was used for all statistical analyses.
